# Supplementary material for: Tau filaments with the chronic traumatic encephalopathy fold in a case of vacuolar tauopathy with VCP mutation D395G
Source: Acta Neuropathol. Author manuscript; Available in PMC 2024 Jun 21. (PMC7616110; doi:10.1007/s00401-024-02741-x)
Supplement: Supplementary Materials [file EMS196676-supplement-Supplementary_Materials.pdf]

SUPPLEMENTARY FIGURE LEGEND

Figure S1

Fourier shell correlation (FSC) curves.

FSC curves of cryo-EM maps (left panel) and model to map validation (right panel). a, CTE Type I tau filament from VT; CTE Type II tau filament from VT; CTE Type III tau filament from VT.

Figure S2

Double-labelling immunofluorescence using anti-tau and anti-glial fibrillary acid protein antibodies.

Sections of frontal cortex from the individual with mutation D395G in *VCP* were labelled with: a, anti-tau antibody AT8 (green) and anti-glial fibrillary acidic protein antibody (red); b, anti-tau antibody pS396 (red) and anti-glial fibrillary acidic protein antibody (green). DAPI nuclear staining is in blue. There was no evidence for the co-localisation of AT8, pS396 and glial fibrillary acidic protein. Scale bar, 50 μm.
